# Comparison of Low Urinary Tract Symptoms during Pregnancy between Primiparous and Multiparous Women

**DOI:** 10.1155/2014/303697

**Published:** 2014-11-09

**Authors:** Kun-Ling Lin, Ching-Ju Shen, Ming-Ping Wu, Cheng-Yu Long, Chin-Hu Wu, Chiu-Lin Wang

**Affiliations:** ^1^Department of Obstetrics and Gynecology, Kaohsiung Medical University Hospital, Kaohsiung Medical University, 100 Shih-Chuan 1st Road, Kaohsiung 80708, Taiwan; ^2^Department of Obstetrics and Gynecology, Chi Mei Foundation Hospital, Tainan 710, Taiwan; ^3^Department of Obstetrics and Gynecology, Kaohsiung Municipal Hsiao-Kang Hospital, Kaohsiung Medical University, 100 Shih-Chuan Road, San-Min District, Kaohsiung 80708, Taiwan

## Abstract

*Background and Purpose.* Low urinary tract symptoms (LUTS) are a common problem during pregnancy. This study aimed to compare changes in the prevalence of LUTS during pregnancy between primiparous and multiparous women. *Methods*. A chart review of consecutive pregnant women who attended our antenatal clinic from March 2002 to January 2006 was performed. All of the women were asked to respond to a LUTS questionnaire in either of the three trimesters. *Results.* Of the 270 women included, 164 were nullipara and 106 were multipara. The most common LUTS during pregnancy were frequency (77%), followed by nocturia (75.6%), stress urinary incontinence (SUI) (51.1%), incomplete emptying (43.7%), dysuria (17.8%), and urgency incontinence (10.4%). There was a significantly higher prevalence of SUI (*P* < 0.001) and urgency incontinence (*P* = 0.005) in the multiparous compared to the nulliparous women. Increasing prevalence rates of frequency, nocturia, SUI, and incomplete emptying were reported with gestational age in both the nulliparous and multiparous women. *Conclusions*. Frequency and nocturia were the two most common LUTS during pregnancy. The prevalence rates of all LUTS increased with increasing gestational age except for frequency in the nulliparous women during the second trimester. In addition, multipara was a predictor of SUI during pregnancy.

## 1. Introduction

Physical and anatomical changes occur in women during pregnancy, including low urinary tract function in the antenatal period. The physiology of low urinary tract symptoms (LUTS) during pregnancy includes multiple factors such as hormonal ef fects, compression of the gravid uterus, and anatomical alterations in pelvic support [1].

Frequency and nocturia are the most commonly reported urinary symptoms during pregnancy. van Brummen et al. [2] analyzed nulliparous women and reported a prevalence of frequency symptoms of 74.2%, with the prevalence remaining high until the third trimester. Moreover, frequency and nocturia were obviously associated with increasing gestational age, and a higher incidence of these symptoms was found in the nulliparous compared to the multiparous group. On the other hand, stress urinary incontinence (SUI) is also a prominent LUTS during pregnancy, with a reported prevalence rate of around 40% [3]. The incidence of SUI is associated with gestational age, parity, and body mass index [4–6]. As gestational age increases, the occurrence of SUI increases, and previous studies have reported that more multiparous women experience SUI than nulliparous women [6–8].

To assess LUTS during pregnancy in Taiwan, Sun et al. [7] analyzed 799 normal pregnant women and found that the most common LUTS were nocturia (60.2%) and SUI (46.1%), followed by urgency (34.1%), frequency (27.8%), incomplete emptying (26.2%), a bearing-down sensation (23.8%), and voiding difficulty (12.6%). The most common LUTS generally increased with increasing gestational age [9], and a higher prevalence of SUI in multiparous women has also been reported [4].

Understanding the changes in LUTS during pregnancy will allow clinicians to provide pregnant women with more complete information and care. Previous studies of LUTS in women during pregnancy have tended to focus on only one symptom such as SUI [10, 11] or irritating symptoms [12]. In this study, we aimed to assess changes in the prevalence of LUTS during the three trimesters of pregnancy and analyze the relationship between primiparous and multiparous women.

## 2. Materials and Methods

A chart review of consecutive pregnant women who attended our antenatal clinic from March 2002 to January 2006 was performed. The Ethics Committee of our university hospital approved the study protocol. The exclusion criteria were urinary tract infections, severe preeclampsia, renal disease, overt diabetes mellitus, and gestational diabetes mellitus. A total of 270 women (164 nulliparous and 106 multiparous) were enrolled in this study.

All of the women underwent a personal interview to evaluate urinary symptoms with a standard questionnaire (as in the appendix) based on the definitions of the International Continence Society in which LUTS can be divided into storage, voiding, and postmicturition symptoms [13]. The questionnaire included questions describing symptoms of SUI, diurnal and nocturnal frequency, urgency incontinence, incomplete bladder emptying, and painful urination (dysuria).

The prevalence of various LUTS in the first trimester (before 12 weeks of gestation), second trimester (13–27 weeks of gestation), and third trimester (after 28 weeks of gestation) was calculated based on the proportion of women reporting the LUTS divided by the total number of women. The answers to all of the questions were either yes or no. The questionnaires were mostly self-administered by the subjects. Data on demographic characteristics including ethnicity, age, education, occupation, body weight before pregnancy, parity, and gestational age were collected from the medical charts. The data were analyzed using the chi-squared test. A *P* value of less than 0.05 was considered to be statistically significant.

## 3. Results

Of the 270 women included, 164 were nulliparous and 106 were multiparous. The average ages of the nulliparous and multiparous subjects were 26.2 years and 29.7 years, respectively (*P* < 0.01). The prevalence rates of LUTS in both groups were evaluated during the three trimesters. The most common LUTS during pregnancy were frequency (77%), followed by nocturia (75.6%), SUI (51.1%), incomplete emptying (43.7%), dysuria (17.8%), and urgency incontinence (10.4%). There was a significantly higher prevalence of SUI (*P* < 0.001) and urgency incontinence (*P* = 0.004) in the multiparous than in the nulliparous women. The rates of frequency and nocturia were more common in the multiparous women compared with the nulliparous women; however, the differences were not statistically significant. In addition, the nulliparous women had higher prevalence rates of incomplete emptying and dysuria than the multiparous women, but again the differences were not statistically significant (Table 1).

**Figure 1:**
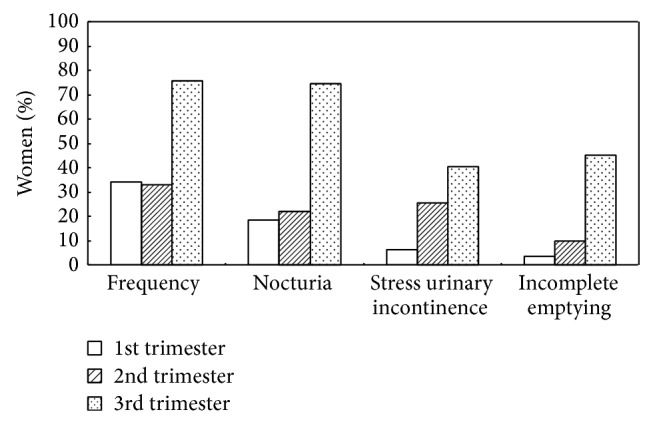
Prevalence of lower urinary tract symptoms during pregnancy by gestational age in nulliparous women (*n* = 164).

**Figure 2:**
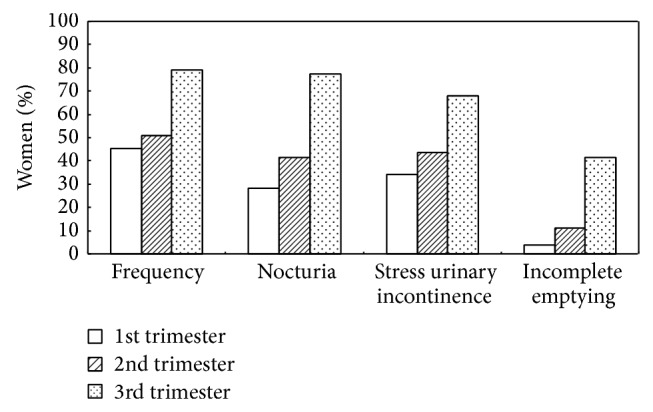
Prevalence of lower urinary tract symptoms during pregnancy by gestational age in multiparous women (*n* = 106).

The multiparous women (41.5%) in the second trimester had more nocturia symptoms than the nulliparous women (22%) (*P* = 0.03). Apart from this finding, a significantly higher rate of SUI during every trimester (first trimester: 34%; second trimester: 43.4%; and third trimester: 67.9%) was found in the multiparous women compared with the nulliparous women (first trimester: 6.1%; second trimester: 25.6%; and third trimester: 40.2%) (*P* < 0.001; *P* = 0.05; and *P* = 0.011, resp.) (Table 2). Increased prevalence rates of frequency, nocturia, SUI, and incomplete emptying were found with increasing gestational age in both the nulliparous and the multiparous groups (Figures 1 and 2).

## 4. Discussion

Previous studies have shown that the prevalence of LUTS such as storage problems with urinary frequency, nocturia, and urgency incontinence is common during pregnancy [2, 7, 10]. In this study, we found that the most prevalent LUTS during pregnancy were frequency and nocturia, followed by SUI and incomplete bladder emptying. These findings are similar to the results of Sun et al. [7]. van Brummen et al. [2] reported a high prevalence of frequency and urgency symptoms at 12 weeks of gestational age and that these symptoms then remained stable during the other two trimesters. However, we obtained opposite results in that irritating symptoms progressively occurred with advanced gestational age, in accordance with previous studies [7, 14].

Frequency, nocturia, and SUI are common urinary problems during pregnancy. Viktrup [15] reported that frequency and nocturia did not significantly increase in the five years after the first delivery. Persistent SUI and urgency urinary incontinence three months after delivery are risk factors for long-lasting problems. Pelvic muscle training has been shown to improve SUI, frequency, and urgency, and therefore antepartum pelvic muscle training is important to prevent postpartum urinary symptoms [16–18].

Our finding that multiparous women experienced more SUI than nulliparous women is similar to previous studies [6–8]. Panayi and Khullar reported that approximately 20% of multiparous women with SUI during the first trimester had levator ani muscle defects on magnetic resonance imaging compared to nulliparous women [19]. Previous pelvic floor trauma after vaginal delivery resulting in poor support for the urethra may explain why multiparous women have a higher prevalence of SUI [20]. In addition, other studies [2, 21] have reported that the course of pregnancy also plays an important role in the pathophysiology of urinary incontinence and especially SUI.

In our study, the prevalence of nocturia increased steadily with gestational age inboth the multiparous and nulliparous women, while (diurnal) frequency increased steadily with gestational age only in the multiparous women. However, two prospective studies [12, 14] found stable increases in the prevalence rates of nocturia and frequency throughout pregnancy in both nulliparous and multiparous women. This discrepancy may partly be due to differences in ethnicity and study design. We found that the rates of nocturia and frequency were most prevalent during the third trimester, possibly due to the compression effect of gravid uterus and more urine output [22, 23].

In this study, nocturia and frequency were the most common LUTS during pregnancy in both the nulliparous and multiparous women. The multiparous pregnant women had higher prevalence rates of frequency and nocturia compared with the nulliparous women, which is comparable to the report of Stanton et al. [14]. Long et al. reported increased mRNA levels of the M3 receptor in the bladder after significant birth trauma in an animal study [24]. This may partly explain why urinary frequency, nocturia, and even urgency incontinence occur more commonly in multiparous women.

Other LUTS explored in this study were less prevalent. For example, we found that the prevalence of incomplete emptying was 43.7% and the prevalence of urge incontinence was 10.4%. These findings are similar to the study of Cutner et al. [25] who reported rates of 26.0% and 10.0%, respectively. Interestingly, we found that the prevalence rates of incomplete emptying and dysuria in the nulliparous women were higher than in the multiparous women, however the mechanism remains unclear.

The results of our study showed that irritating symptoms such as frequency and nocturia were the most common LUTS during pregnancy, followed by SUI. A significantly higher rate of SUI during all three trimesters was found in the multiparous compared with nulliparous women; that is, being multiparous was a predictor of SUI during pregnancy. The prevalence rates of all LUTS increased with increasing gestational age except for urinary frequency in the nulliparous women during the second trimester (Table 3). A limitation of this study is that we did not use a three-day voiding diary to evaluate the frequency and severity of LUTS during pregnancy nor investigate postpartum status. Further studies are necessary to explore LUTS during pregnancy with more detailed measurements and follow-up for a significant period of time, which may reveal further predictors for LUTS to help prevent their postpartum occurrence.

## Figures and Tables

**Table 1:** The clinical background in nulliparous and multiparous women. Data are given as mean ± standard deviation or percentage.

Symptoms	Nulliparous (*n* = 164)	Multiparous (*n* = 106)	*P* value
Age (years)	29.3 ± 3.3	32.1 ± 5.9	<0.001^a^
Education level			
Primary school	2.4%	3.9%	0.25^b^
Senior high school	32.5%	34.2%	0.33^b^
College	65.1%	61.9%	0.18^b^
Body height (cm)	158.3 ± 6.4	157.8 ± 4.9	0.11^a^
Modes of previous delivery			
Vaginal delivery		67.7%	
Cesarean section		33.3%	
Employment	45.5%	39.8%	0.09^b^

^a^Student's *t-*test; ^b^chi-square test.

**Table 2:** The prevalence of lower urinary tract symptoms during pregnancy in nulliparous and multiparous. Data are given as *n* (percentage).

Symptoms	Nulliparous (*n* = 164)	Multiparous (*n* = 106)	Total (*n* = 270)
Frequency	124 (75.6)	84 (79.3)	208 (77.0)
Nocturia	122 (74.4)	82 (77.4)	204 (75.6)
Stress urinary incontinence	66 (40.2)^a^	72 (68)^a^	138 (51.1)
Incomplete emptying	74 (45.1)	44 (41.5)	118 (43.7)
Urge incontinence	10 (6.1)^b^	18 (17)^b^	28 (10.4)
Dysuria	36 (22.0)	12 (11.3)	48 (17.8)

^a^ *P* < 0.001; ^b^ *P* = 0.004; chi-square test. Other symptoms did not reach statistical significance.

**Table 3:** Comparisons of lower urinary tract symptoms by gestational age in nulliparous (*n* = 164) and multiparous women (*n* = 106). Data are given as *n* (percentage).

Symptoms	First trimester		Second trimester		Third trimester	
	Nulliparous	Multiparous	Nulliparous	Multiparous	Nulliparous	Multiparous
Frequency	28 (34.1)	24 (45.3)	27 (32.9)	27 (50.9)	62 (75.6)	42 (79.2)
Nocturia	15 (18.3)	15 (28.3)	18 (22)	22 (41.5)^a^	61 (74.4)	41 (77.4)
SUI	5 (6.1)^b^	18 (34)^b^	21 (25.6)^c^	23 (43.4)^c^	33 (40.2)^d^	36 (67.9)^d^
IE	3 (3.7)	2 (3.8)	8 (9.8)	6 (11.3)	37 (45.1)	22 (41.5)
UI	0	1 (1.9)	0	2 (3.8)	5 (6.1)	9 (17.0)
Dysuria	0	2 (3.8)	0	3 (5.7)	18 (22.0)	6 (11.3)

SUI: stress urinary incontinence; IE: incomplete emptying; UI: urge incontinence; ^a^ *P* = 0.03; ^b^ *P* < 0.001; ^c^ *P* = 0.05; ^d^ *P* = 0.011; chi-square test. Other symptoms did not reach statistical significance.

## Appendix

Lower urinary tract symptoms questionnaire during pregnancy is as follows.

(1) Do you have urine leakage when you
  (a) cough, laugh, sneeze, or during physical exercise,
  (b) do housework, walk, or change position? (stress urinary incontinence)
(2) Do you go to the toilet more than seven times during the day? (frequency)
(3) Do you have to wake up to void more than once during the night? (nocturia)
(4) Do you leak urine if you have to wait to use the toilet or leak on your way to the toilet? (urgency incontinence)
(5) Do you feel as though you have incomplete bladder emptying after rising from the toilet? (incomplete emptying)
(6) Do you have a painful sensation during urination? (dysuria).
